# Reactive Recruitment of Attentional Control in Math Anxiety: An ERP Study of Numeric Conflict Monitoring and Adaptation

**DOI:** 10.1371/journal.pone.0099579

**Published:** 2014-06-11

**Authors:** Macarena Suárez-Pellicioni, María Isabel Núñez-Peña, Àngels Colomé

**Affiliations:** 1 Department of Behavioral Sciences Methodology, Faculty of Psychology, University of Barcelona, Barcelona, Spain; 2 Institute for Research on the Brain, Cognition and Behavior (IR3C), University of Barcelona, Barcelona, Spain; 3 Department of Basic Psychology, Faculty of Psychology, University of Barcelona, Barcelona, Spain; University of Akron, United States of America

## Abstract

This study uses event-related brain potentials (ERPs) to investigate the electrophysiological correlates of numeric conflict monitoring in math-anxious individuals, by analyzing whether math anxiety is related to abnormal processing in early conflict detection (as shown by the N450 component) and/or in a later, response-related stage of processing (as shown by the conflict sustained potential; Conflict-SP). Conflict adaptation effects were also studied by analyzing the effect of the previous trial’s congruence in current interference. To this end, 17 low math-anxious (LMA) and 17 high math-anxious (HMA) individuals were presented with a numerical Stroop task. Groups were extreme in math anxiety but did not differ in trait or state anxiety or in simple math ability. The interference effect of the current trial (incongruent-congruent) and the interference effect preceded by congruence and by incongruity were analyzed both for behavioral measures and for ERPs. A greater interference effect was found for response times in the HMA group than in the LMA one. Regarding ERPs, the LMA group showed a greater N450 component for the interference effect preceded by congruence than when preceded by incongruity, while the HMA group showed greater Conflict-SP amplitude for the interference effect preceded by congruence than when preceded by incongruity. Our study showed that the electrophysiological correlates of numeric interference in HMA individuals comprise the absence of a conflict adaptation effect in the first stage of conflict processing (N450) and an abnormal subsequent up-regulation of cognitive control in order to overcome the conflict (Conflict-SP). More concretely, our study shows that math anxiety is related to a reactive and compensatory recruitment of control resources that is implemented only when previously exposed to a stimuli presenting conflicting information.

## Introduction

The anxiety towards mathematics has been defined as a “feeling of tension and apprehension surrounding the manipulation of numbers and the solving of mathematical problems in academic, private and social settings” [1]. This type of anxiety has been attracting considerable research interest in recent years given that its negative impact on students’ mathematical development is becoming increasingly clear. In this respect, math anxiety is one of the main causes of math avoidance, the tendency of these students to avoid courses and career paths that are related to numbers, a response that stops their mathematical learning at an earlier stage as compared to their low math-anxious counterparts [2]. Undoubtedly this fact has its negative consequences on their professional development, employment opportunities, and even salary prospects.

Beyond these educational and social effects of math anxiety, several investigations have shown that a high math-anxious brain does not work like a low math-anxious one. For example, it has been demonstrated that high math-anxious individuals show: less precise representations of numerical magnitudes [3]; difficulties in counting objects in a visual enumeration task [4]; difficulties in solving complex arithmetic problems [5]; difficulties in processing large-split solutions in simple arithmetic verification [6]; greater cognitive effort and resource investment in preparation for a task goal [7]; abnormal error monitoring for errors committed in a numerical task [8], etc.

The Attentional Control Theory [9] (henceforth ACT), based on the processing efficiency theory [10] (henceforth PET), is one of the main theories trying to explain the negative effects of anxiety on cognitive performance. The original distinction between performance effectiveness (quality of task performance) and processing efficiency (relationship between effectiveness and the amount of resources or effort spent on solving the task), as well as the claim that anxiety affects the latter to a greater extent than the former, are central to ACT. This theory uses the working memory model proposed by Baddeley [11], comprising a central executive (i.e., a modality-free system that controls incoming information) and two slave systems. In this theory, the functions of the central executive are impaired by anxiety, with the inhibition function being one of the most affected [12]. More concretely, according to this theory, anxiety alters the balance between the stimulus-driven attentional system and the top-down goal-driven attentional system [13], reducing the influence of the latter. As a result, high anxious individuals are more easily distracted as compared to low anxious ones. Nevertheless, anxious individuals are considered to compensate for this reduction in efficiency by means of a reactive recruitment of additional attentional resources if these are available.

In this line, Braver and colleagues’ dual mechanisms of control (DMC) theory [14] accounts for two ways of exerting cognitive control that would be associated with the level of anxiety. On the one hand, low anxious individuals are considered to engage top-down control in a *proactive* way, which implies a sustained representation of task requirements or goals. This type of control would allow for more effective top-down control of processing and would promote preparatory attentional and response biases and the prevention of conflict during ongoing processing. By contrast, high anxious individuals are considered to exert control in a *reactive* way, consisting of an only-when-needed late correction character. This type of control implies that, after task goals are first coded, they are not maintained in a continuously active state. In other words, task representations are reactivated only when a task-relevant stimulus is encountered or conflict occurs in processing. This entails weaker preparatory attentional biases, and processing is therefore more easily influenced by bottom-up input. As a consequence, high anxious individuals would be more easily distracted than their low anxious counterparts.

Cognitive control effects have traditionally been measured using the Stroop task. In the original Stroop color-naming task, introduced more than 75 years ago [15], color words are presented in varying colors, and the participant is asked to name the color of the ink (target dimension) while ignoring the word meaning (distractor dimension). An incongruent target-distractor pairing (e.g., the word RED written in blue ink) induces a stimulus-response conflict as compared with congruent target-distractor pairings (e.g., the word RED written in red ink). The *Stroop interference effect* consists of an increase in response times in incongruent trials compared with congruent ones, and has been suggested to show the difficulty in inhibiting attention to meaningful but conflicting information, even when that information is not relevant for solving the task [16].

Following the pioneering research of Stroop (1935), the Stroop interference effect has also been observed using numbers. There are two main numerical Stroop paradigms: one (also called counting task) in which the numerical magnitude denoted by the Arabic digits interferes with saying how many of them there are (e.g., having to say “four” to 3333) [17], [18], and another in which the physical size of the digit interferes with its numerical magnitude or vice versa (e.g., 2 8) [19]. Similarly to their performance on the classic Stroop task, individuals performing the numerical Stroop task take longer and commit more errors when responding to incongruent (e.g., 3333 or 2 8) than to congruent (e.g., 333 or 2 8) trials (i.e., the *numerical interference effect*).

Given the ability of this task for measuring conflict and inhibitory processing, it seems very suitable for assessing the negative effects of anxiety. For example, using a classic Stroop task, Pallak et al. (1975) found that high anxious individuals showed slower response times in the condition presenting conflicting information, that is, in incongruent trials, as compared to the low anxious ones [20]. Similarly, using the same task, another researcher found that individuals in the high-stress condition performed significantly worse than the ones in the low-stress condition, but only for incongruent trials [21], [22].

Despite the relative infancy of math anxiety research, the susceptibility of high math-anxious (HMA) individuals to distraction has already been tested [17], [23]. Hopko et al. (1998) formed three groups of participants according to their level of math anxiety (low, medium, and high) and administered a task designed to measure their ability to inhibit attention to distracting phrases in a reading task. Reading conditions consisted of paragraphs that were categorized by content (i.e., math or non-math) and distractor type (i.e., control, related, and unrelated). Related distractors were math words that were unrelated to paragraph content, unrelated distractors were non-math words also unrelated to paragraph content and, finally, control distractors were a string of Xs, equivalent in length to the other types of distractors, and inserted in the same locations as distracters in the other two conditions. They found that HMA individuals took significantly longer to read paragraphs with distractors embedded in the text than did low math-anxious (LMA) participants. Nevertheless, this slowdown was also shown when paragraphs were unrelated to mathematics, which was taken as evidence supporting HMAs’ difficulty in inhibiting attention to any kind of distractor. Some years later, Hopko et al. (2002) measured those difficulties in attention inhibition in math anxious individuals by using the counting version of the numerical Stroop task. To this end, they formed two groups according to participants’ level of math anxiety (top and bottom 20% of the distribution). Participants were administered a card version of the numerical Stroop task containing both numerical (e.g., 9999) and non-numerical (e.g., HHHH) materials. Participants’ task consisted in saying the quantity of elements (numbers or letters) on each card. In the case of the numerical material, the stimuli were always incongruent. They found that the HMA group showed longer response times with both the numerical and the non-numerical materials, as compared to the LMA group. Nevertheless, this slowdown was significantly higher for the task including numerical material than for the one including letters. The authors interpreted their results in line with previous research [23], [24], suggesting that HMA individuals may possess a more trait-like inability to suppress attention to distracting information, a deficit that seemed not to depend on, but to be somehow enhanced by exposure to numerical stimuli.

Although interference effects in math anxiety have previously been shown in behavioral measures, they have never been studied using more sensitive techniques. For this reason, the main objective of this study was to investigate interference effects in math anxious individuals by means of the event-related potentials (ERPs) technique, which provides a measure of brain dynamics with high temporal resolution, allowing a characterization of the cascade of processes that behavioral measures cannot offer. In this respect, conflict-related effects have been found at very early stages of processing, like the P1 component. The P1 component is a positive-direction component appearing at the parieto-occipital electrodes between 100 and 150 ms post-stimulus which is thought to reflect processing of the low-level features of stimuli [25]. Previous authors have hypothesized that it is generated in posterior occipito-temporal areas [26] and is influenced by amygdala in fear processing [27]. Using compound stimuli consisting of a facial expression with an expressive body, Meeren et al. (2005) found a larger P1 ERP component at posterior brain sites when the expression of the face and the emotion portrayed by the body conflicted than when they were congruent [28].

Despite conflict-related findings for the P1 component, the N450 component and the conflict sustained potential (henceforth Conflict-SP) [18], [29]–[35], consistently identified in the incongruent minus congruent differences wave, are the main ERP components associated with conflict processing. The N450 component is a negative-going ERP deflection appearing from approximately 350 to 500 ms post-stimulus at fronto-central sites. Recent evidence has suggested that this component is related to stimulus conflict processing (i.e., at the level of stimulus representation) rather than to response conflict processing (i.e., at the level of motor response organization) [19]. Source analysis indicates that the neural generators of N450 may lie within the anterior cingulate cortex (ACC) [29], [32], which supports the suggestion that this component reflects conflict detection [18], [32], [36]. Moreover, it shows greater negativity when the level of conflict increases (e.g., reducing the proportion of incongruent stimuli) [31], consistent with previous evidence pointing to an increase in ACC activity in high conflict situations [37].

The N450 is directly followed by a positive-going Conflict-SP, emerging at central sites roughly 500 ms after stimulus onset [35]. Its sources have been suggested to be located within the middle or inferior frontal gyrus (LPFC) and the left extrastriate cortices [31]. The cognitive processes underlying this component are more ambiguous in the literature than those of N450, but they have been associated with general preparation [33], conflict resolution [31], [32], response selection [18], and the execution of top-down control [38]. Their amplitude also varies with the level of conflict, being more positive for high conflict conditions (i.e., when incongruent stimuli are presented in lower proportion) as compared to low conflict ones [39].

Beyond conflict monitoring, another way to study possible deficits in conflict processing is through studies of conflict adaptation (also referred to as sequential-trial effects, trial-to-trial effects, or Gratton effects) [40]. Gratton et al. (1992) observed that, apart from the expected main effect of congruence of the current trial (i.e., longer response time and error rates for incongruent as compared to congruent trials), there was an interaction between current and previous trial congruence, in which the interference was higher following congruent trials than following incongruent ones. The conflict monitoring model (CMM) holds that the conflict adaptation effect stems from conflict-driven adjustments in cognitive control [41]. When an incongruent trial is presented, a simultaneous activation of competing responses (response conflict) is produced. This conflict is detected by a conflict-monitoring mechanism, thought to reside in the anterior cingulate cortex (ACC), which triggers an up-regulation in cognitive control, thought to be implemented by the lateral prefrontal cortex (LPFC), in order to overcome the conflict. Activation in the ACC, reflected in N450, and subsequent activation in the LPFC and left extrastriate cortices, reflected in Conflict-SP, are consistent with the theory that the ACC and prefrontal regions are involved in evaluative processes and subsequent strategic adjustment in attentional control to reduce future conflict [39], [42]–[44]. As a consequence, the level of cognitive control is high following an incongruent trial. In contrast, congruent trials are not associated with response conflict and do not result in a temporary up-regulation of cognitive control. Hence, the level of control is low following a congruent trial.

Regarding ERPs, the N450 component has been suggested not to be influenced by the congruence of the previous trial, that is, not to exhibit a significant conflict adaptation effect. Consequently, it has been considered to reflect a more automatic conflict monitoring mechanism that would not be influenced by the implementation of top-down control [38]. However, recent evidence in the field of anxiety has found variations in this component according to the congruence of the previous trial, and thus has suggested that this component reflects more than an automatic process [45]. On the other hand, Conflict-SP has also been shown to index previous-trial congruence, showing greater amplitude for the interference effect preceded by congruence (*cI-cC*) than for the interference effect preceded by incongruity (*iI-iC*). The greater amplitude of Conflict-SP when preceded by congruence implies a higher level of interference (the greater the amplitude, the greater the interference), given that attentional control is considered not to be enhanced by the preceding congruent trial. On the contrary, a reduction in its amplitude when preceded by incongruity trials has to do with a reduced level of interference, given that an enhanced attentional control is considered to have been exerted in the preceding incongruent trial. This evidence suggests that the amplitude modulations of this Conflict-SP reflect conflict adaptation effects, that is, controlled processes signaling for increased implementation of attentional control after conflict detection [38], [39], [46].

Previous evidence has shown that trait anxiety is closely related to individual differences in dynamic adjustments of attentional control, supporting the association between high anxiety and a reactive use of attentional control suggested by the DMC account [14] and the ACT [9]. As commented above, Osinsky et al. (2010), using a gender discrimination Stroop task (This task consists of the presentation of male and female faces together with the word “woman” or “man”, which results in congruent trials (e.g., a woman’s face with the word “woman”) and incongruent trials (e.g., a man’s face with the word “woman”) and participants have to respond to the gender of the face, while the word acts as a distractor), found a more negative deflection in the N450 time window in the context of preceding incongruent trials as compared to preceding congruent trials for the high trait-anxious group, suggesting that these individuals more strongly engage neural mechanisms of conflict-monitoring only when previously exposed to a high level of stimulus-response conflict (i.e., only after incongruent trials) [14], [47], [48]. Some years later, the same research group performed a similar experiment using the same gender discrimination task (face-word pairings) but incorporating trials where only the relevant dimension of the task was presented (face-only trials) and others where only the task-irrelevant dimension of the task was shown (word-only trials) [45]. For the face-word and the face-only stimuli, participants were instructed to discriminate the sex of the presented faces, while they were instructed to react to the word meaning of the word-only stimuli. The N170 and N400, two ERPs components related to face and word processing, respectively, were analyzed. They found that high trait-anxious participants showed a higher N170 component for face-only trials when preceded by incongruent face-word pairings, signaling faster face discrimination after conflict processing, and higher N400 for the word-only condition, suggesting slower word discrimination, and thus suppressed processing of the task-irrelevant dimension of the task. They interpreted their results as evidence suggesting that high trait anxiety is linked to a reactive and compensatory recruitment of attentional control resources following a conflict between task-relevant and task-irrelevant stimuli, as previously suggested by other authors [14], [47].

As we noted previously, although susceptibility to distraction in math anxious individuals has been studied previously by means of behavioral measures [17], [23] no work to date has investigated its electrophysiological correlates. Studying numeric interference by means of the sensitive ERP technique would allow us to identify two main conflict-related ERP components, N450 and the subsequent Conflict-SP, and thus to further investigate whether math anxiety is related to an earlier conflict detection and/or to a later response-related stage of processing. Similarly, conflict adaptation effects in math anxiety have never been studied. Since neural and behavioral evidence of conflict adaptation is sensitive to subtle differences in cognitive processing, it can be especially useful for identifying the specific nature of cognitive processing deficits in math anxious individuals when they have to deal with conflicting information.

With these objectives in mind, we formed two groups that were extreme in their level of mathematical anxiety (top and bottom 25% of the distribution forming the HMA and LMA groups, respectively). Groups did not differ in trait or state anxiety or in math ability, in order to rule out the possibility that any group differences could be explained by differences in these variables. Participants performed a single-trial version of the numerical Stroop task presenting conflict between numerical magnitude and physical size, while their ongoing EEG was recorded. Two main effects were analyzed: conflict monitoring effects and conflict adaptation effects. While the former analysis assessed the congruence effect of the current trial, the latter studied modulations in attentional control for the current interference effect depending on the congruence of the previous trial. In the case of the first main effect, the conflict monitoring analysis was performed by comparing the N450 and Conflict-SP components between groups for the interference effect (incongruent-congruent difference wave). It has been suggested that it is very difficult to measure the amplitude and latency directly from a raw ERP waveform without distortion from overlapping components. For this reason, creating difference waves can constitute a good strategy for isolating the component of interest [49]. In the case of the second main effect, the conflict adaptation analysis was performed by comparing the same ERP components between groups for the interference effect preceded by congruence (*cI-cC*) and by incongruity (*iI-iC*). Our hypotheses were as follows. Regarding the conflict monitoring analysis, we expected: 1) to reproduce previous findings on math anxiety [17], [23], by obtaining a higher interference effect (incongruent-congruent) in response times for the HMA group as compared to the LMA one. Differences were expected for response times and not for error rates given that, according to the ACT, behavioral consequences of anxiety-related deficits would affect response time (i.e., processing efficiency) but not accuracy (i.e., performance effectiveness) [9]. 2) Regarding ERPs, as suggested by previous evidence, conflict-related brain potentials should increase with the level of anxiety [45], so we expected greater N450 and/or Conflict-SP amplitudes for the HMA group as compared to the LMA group. As for the conflict adaptation analysis, we expected: 3) to find the conflict adaptation effect for the two groups, with the interference effect expected to be smaller when preceded by incongruity than when preceded by congruence [44] given that incongruity in the previous trial would have enhanced attentional control and thus would have reduced the influence of the distractor. No differences between groups were expected for this conflict adaptation effect in behavioral measures, as suggested by previous evidence analyzing this effect in trait anxiety [48]. 4) Differences between groups were expected to be found in ERPs though. Given that there is no clear evidence for conflict adaptation modulations for the N450 component, with some authors suggesting that it reflects a more automatic conflict monitoring mechanism, not influenced by variations in attentional control [38] and another study reporting a modulation of the N450 component by previous trial congruence in trait anxiety [48], no clear hypothesis were formulated for this component. On the contrary, conflict adaptation effects were expected for the Conflict-SP, a component clearly linked to the execution of top-down control [18], [29], [32], [38]. Thus, if, as suggested by the ACT [9] and the DMC [14], anxiety is related to a reactive recruitment of attentional control [45], [48], then the HMA group would exert attentional control only after incongruent trials (i.e. when conflict is encountered in processing), so they should show a reduced Conflict-SP for the interference effect preceded by incongruity (*iI-iC*) as compared to the interference effect preceded by congruence (*cI-cC*) (i.e. the greater the conflict, the greater the Conflict-SP). On the other hand, the LMA group, considered to engage top-down control in a proactive or sustained way, should show no difference in the Conflict-SP component for the interference effect depending on the congruence of the previous trial.

## Methods

### Participants

Thirty-four healthy volunteers were tested in this study, half of them with a high level of math anxiety (HMA) and the other half with a low level (LMA). They were selected from a sample of 490 university students from the University of Barcelona who were assessed for math anxiety, trait and state anxiety and simple math ability.

The LMA group comprised seventeen participants (age range = 19–26, mean = 21.18, SEM = .50), who scored below the first quartile in the Abbreviated Mathematics Anxiety Rating Scale (sMARS) [50] (score range = 35–52, mean = 45.76, SEM = 1.22). The HMA group also comprised seventeen participants (age range = 19–25, mean = 20.82, SEM = .41), but these scored above the third quartile in the sMARS (score range = 76–102, mean = 85.29, SEM = 1.61). More detailed information about the two groups is shown in Table 1.

**Table 1:** Means and standard errors of the mean (SEM; in brackets) for age, educational level, math ability, math anxiety, trait and state anxiety and frequencies for gender and handedness for the LMA and the HMA groups.

	Age	Gender	Handedness	Education	Ability	sMARS	STAI-T	STAI-S
LMA	21.18 (.50)	13	16	15.65 (.44)	.98 (.004)	45.76 (1.22)	20.06 (2.56)	14.24 (8.21)
HMA	20.82 (.41)	14	16	15.00 (.30)	.98 (.006)	85.29 (1.61)	22.53 (2.68)	19.18 (8.97)

Note: LMA: low math-anxious; HMA: high math-anxious; Gender: number of women; Handedness: number of right-handed individuals; Education: number of years of formal education counting from 6 years-old forward. Ability: proportion of correctly solved additions with respect to the total of additions solved in the Simple arithmetic test. sMARS: shortened Math Anxiety Rating Scale; STAI-T: Trait anxiety subscale from the STAI; STAI-S: State anxiety subscale from the STAI.

Groups differed in math anxiety (*t*(32) = 19.49, *p*<.001), but not in trait anxiety (*t*(32) = .66, *p = *.51), state anxiety (*t*(32) = 1.67, *p* = .11), simple math ability (*t*(31) = .54, *p* = .59), age (*t*(32) = .53, *p = *.59), years of formal education (*t*(32) = 1.19, *p = *.24), handedness (*χ^2^ = *.00, *p = *1), ethnicity (*χ^2^* = 1.03, *p = *.31) or gender distribution (*χ^2^* = .18, *p = *.67).

All participants had normal or corrected-to-normal visual acuity and did not report any history of neurological or psychiatric disorders. All were naïve as to the purposes of the study.

### Ethics Statement

Participants were paid for their participation and gave written informed consent before the experiment. The experimental protocol was approved by the Bioethics Committee of the University of Barcelona and was in accordance with the Code of Ethics of the World Medical Association (Declaration of Helsinki).

### Materials

#### During the screening phase of the study

The following tests were administered in order to form groups. They were presented to the participant in the following order:

Simple Arithmetic Test: This test consists of 165 single-digit addition problems of the form “a+b = ” organized into five columns. There were 24 different additions involving operands between 2 and 9. No addition included the numbers 1 or 0 or tie problems (i.e. 4+4). Individuals were instructed to solve the additions as fast and as accurately as possible within a time limit of two minutes. This test has been previously used for measuring simple arithmetic ability in another study performed by our lab [6]. Given the simplicity of the task (the most difficult addition was 8+9 = ), the accuracy in solving it (the proportion of correctly solved additions with respect to the total of additions solved) was taken as a measure of participants’ simple arithmetic ability.

Abbreviated Mathematics Anxiety Rating Scale (sMARS) [50]: The sMARS is a 25-item version of the Math Anxiety Rating Scale (MARS) [1]. This instrument measures anxiety by presenting 25 situations which may cause math anxiety (e.g., *Being given a homework assignment of many difficult problems that are due in the next class meeting*). Participants decide on the level of anxiety associated with each item by answering on a five-point Likert scale from 1 (no anxiety) to 5 (high anxiety). The sum of the item scores provides the total score for the instrument, which ranges from 25 to 125. In the present study, the Spanish version of the sMARS [51] was used. The scores for the Spanish version of the sMARS have shown strong internal consistency (Cronbach’s alpha = .94) and high 7-week test-retest reliability (intra-class correlation coefficient = .72).

State-Trait Anxiety Inventory (STAI) [52]: It includes 40 statements describing different emotions, 20 for measuring state anxiety (STAI-S) and 20 for trait anxiety (STAI-T). Items are answered on a four-point Likert scale. In the STAI-S the answer options go from 0 (not at all) to 3 (very much) and subjects have to answer by taking into account how they feel “right now”. In the STAI-T the answer options go from 0 (rarely) to 3 (almost always) and subjects have to answer by taking into account how they feel “in general” [52]. Good to excellent internal consistency (Cronbach’s alpha = .86–.95) and adequate test–retest reliability (State: *r* = .71–.76; Trait: *r* = .75–.86) has been reported [52]. The Spanish version of this test has been used in this study, which also has shown good psychometric properties [53].

#### During the recording session

Participants were administered a numerical Stroop task comprising pairs of Arabic numbers (1–2, 1–8, 2–9 and 8–9) shown simultaneously in the middle of the computer screen. Numbers were presented in two sizes: large (font 80) and small (font 40). Stimulus pairs appeared at subtended viewing angles of 0.68° and 1.37° (horizontally) and 0.97° and 1.77° (vertically) for large and small sizes, respectively. Participants were asked to respond to the number of higher numerical magnitude, ignoring physical size. The stimuli could be congruent (the number of larger numerical magnitude was also larger in physical size; e.g., 8 9) or incongruent (the number of larger numerical magnitude was smaller in physical size; e.g., 8 9) [54]. The task included congruent and incongruent stimuli in equal proportions and all the stimuli were presented an equal number of times and randomly to each participant.

Participants were instructed to indicate the number of larger numerical magnitude by clicking on the left or right button of the mouse, depending on the side of the screen in which it had appeared. The side on which the larger number appeared was counterbalanced, so there were two instances for all number pairs (e.g., 8 9 and 9 8). They were asked to respond as fast and as accurately as possible.

The E-prime 2.0 program (Psychology Software Tools Inc., Sharpsburg, PA, USA) was used to control the presentation and timing of the stimuli and to measure response accuracy and response time.

### Procedure

Participants were tested individually. Upon entering the experimental room, they completed standard procedures concerning informed consent along with a demographic questionnaire asking their age, ethnicity, gender, and number of years of formal education. Then, EEG/EOG sensor electrodes were attached and the participant was given detailed task instructions. After that, participants were seated 100 cm away from a computer screen in an electrically-shielded, sound-attenuating recording chamber. The experimental session began with a training period of 24 trials. When participants achieved 65% of hits in the training period, the recording session started (if not, the training was repeated). The training trials were used only to familiarize the participants with the task, so they were excluded from the statistical analysis.

**Figure 1:**
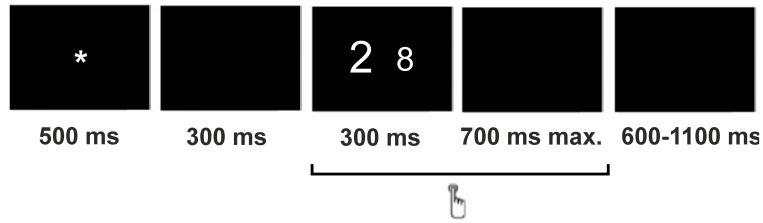
Structure and timing of a trial of the numerical Stroop task using an incongruent stimulus.

Each trial began with a fixation sign (an asterisk) shown for 500 ms. After a 300 ms pause (black screen), a pair of numbers were shown for 300 ms and then followed by a 700 ms-black screen (maximum response window of 1000 ms). Each trial was followed by a variable inter-trial interval ranging from 600 to 1100 ms (black screen). Participants responded to 160 total trials, 80 per condition, organized into 5 blocks of 32 stimuli and preceded by the 24 practice stimuli. The whole session lasted about 120 minutes. Figure 1 shows the sequential presentation of an incongruent stimulus and its timing.

### Electrophysiological Recording

The EEG was recorded with ANT hardware and software (B.V., Enschede, The Netherlands) from 64 electrodes mounted in a commercial WaveGuard EEG Cap (Eemagine Medical Imaging Solutions GmbH. ANT Advanced Neuro Technology) and positioned according to the extended 10/20 system, as well as two electrodes on the right and left mastoids. EEG channels were continuously digitized at a rate of 512 Hz by an ANT amplifier (B.V., Enschede, The Netherlands). A band-pass filter was set from 1.6 to 30 Hz, and electrode impedance was kept below 5 kΩ. The horizontal and vertical electrooculogram was recorded with electrodes placed at the outer canthus and below the right eye, respectively. The common reference electrode was placed on the tip of the nose and the ground was located at AFz. For data analysis, they were re-referenced to the mean activity of all sites [55]. Ocular artifacts were identified and corrected with the eye-movement correction algorithm used in the EEprobe program (ANT, The Netherlands). For graphical presentations only, a 15-Hz low-pass filter was applied.

## Data Analysis and Results

### Behavioral Data

#### Conflict monitoring analysis

Medians of response times (RT) for correctly solved trials and percentage of hits were calculated for each participant in each condition (congruent and incongruent). Following previous studies, we calculated a single score index of interference by subtracting congruent from incongruent trial latencies for the RT analysis and incongruent from congruent hit rates in the accuracy one (i.e. for both indices, the greater the value, the greater the interference) [48]. A *t* test was carried out to look for group differences in the interference effect.

Regarding response times, significant differences were found between groups (*t*(32) = 2.10, *p* = .04), with the HMA group showing a greater interference effect (mean = 72.50 ms, SEM = 8.15) than the LMA one (mean = 52.02, SEM = 5.30).

No significant differences were found for percentage of hits (*t*(32) = .44, *p* = .66).

**Conflict adaptation analysis.** Medians of response times for correctly solved trials and percentage of hits were calculated for each participant in each condition: incongruent trials preceded by congruence (*cI*), congruent trials preceded by congruence (*cC*), incongruent trials preceded by incongruity (*iI*) and congruent trials preceded by incongruity (*iC*). Then, these means were used to calculate the interference effect preceded by congruence (*cI-cC*) and the interference effect preceded by incongruity (*iI-iC*). Similarly, hit rates were calculated for the interference effect preceded by congruence (*cC-cI*) and preceded by incongruity (*iC-iI*). A potential confound of examining the neural and behavioral reflections of conflict adaptation effects is the inclusion of error and post-error trials [42]. Error trials are frequently associated with faster RTs [56], while post-error trials are associated with reliable RT slowing [57]. In order to separate the effect of error processing from the conflict adaptation processes, error and post-error trials were excluded from both the conflict monitoring and the conflict adaptation analyses.

Response time and hit rate data were submitted to a repeated measures ANOVA taking *Previous congruence* (congruent and incongruent) as the within-subject factor and *Group* (LMA and HMA) as the between-subjects factor. The *F* value, the degrees of freedom, the probability level, and the *η^2^* effect size index are given.

Regarding response times, the ANOVA showed a significant main effect of *Previous congruence* (*F*(1,32) = 4.16, *p = *.04, *η^2^* = .11), with the interference effect being higher when preceded by congruence (mean = 64.57, SEM = 5.89) than when preceded by incongruity (mean = 51.33, SEM = 5.48). The main effect of *Group* was also significant (*F*(1,32) = 4.15, *p = *.05, *η^2^* = .11), showing that, regardless of the congruence of the previous trial, the HMA group was slower (mean = 67.50, SEM = 6.61) than the LMA one (mean = 48.41, SEM = 6.61). The *Previous congruence* × *Group* interaction was far from significant (*p* = .64).

As for percentage of hits, the ANOVA showed a significant main effect of *Previous congruence* (*F*(1,32) = 5.31, *p = *.02, *η^2^* = .14), with the interference effect being higher when preceded by congruence (mean = 21.64, SEM = 2.30) than when preceded by incongruity (mean = 17.22, SEM = 2.25). The main effect and interactions with *Group* were far from significant (all *p* values above.57).

Response times and percentage of hits for each group for the conflict monitoring and conflict adaptation effects are shown in Table 2.

**Table 2:** Response times (mean of medians) and accuracy (percentage of hits) (SEM in brackets) for the LMA and HMA groups for conflict monitoring and for conflict adaptation effects.

		Conflict monitoring	Conflict adaptation	
		Interference	Interference preceded by congruence	Interference preceded by incongruity
Response time	LMA	52.02 (5.30)	56.52 (7.13)	40.29 (5.26)
	HMA	72.50 (8.15)	72.61 (9.37)	62.38 (9.62)
Hit rates	LMA	21.62 (2.83)	23.08 (3.36)	17.56 (2.69)
	HMA	20.00 (2.32)	20.22 (3.13)	16.89 (3.61)

Note. Conflict monitoring: for response time: interference = incongruent – congruent; for hit rates: interference = congruent – incongruent. Conflict adaptation: for response time: interference preceded by congruence = *cI-cC*; interference preceded by incongruity = *iI-iC;* for hit rates: interference preceded by congruence =  *cC-cI*; interference preceded by incongruity = *iC-iI.*

### Event-Related Potentials

ERPs time-locked to the presentation of the stimuli were averaged for each participant. As in the behavioral analysis, error and post-error trials were not included in the analysis. The averages were constructed from −100 to 1000 ms epochs relative to stimulus onset. A 100-ms window prior to the stimulus (−100 to 0 ms) served as the baseline. Trials with voltages exceeding ±75 µV in any electrode were excluded from the ERP average. Only trials correctly answered were included. For the conflict monitoring analysis, two averages were calculated per participant: one for congruent trials and another for incongruent trials. As in previous investigations [19], [29], [32], [35], [58], interference was defined as the incongruent minus the congruent conditions. For the conflict adaptation analysis, four averages were calculated per participant: incongruent trials preceded by incongruity (*iI*), incongruent trials preceded by congruence (*cI*), congruent trials preceded by incongruity (*iC*), and congruent trials preceded by congruence (*cC*). The interference effect preceded by congruence was calculated by subtracting the cC trials from the cI trials (*cI-cC*), while the interference effect preceded by incongruity was calculated by subtracting the iC trials from the *iI* trials (*iI-iC*).

#### Conflict monitoring analysis

For all the ANOVAs performed in this study, the Greenhouse-Geisser correction [59] for violations of sphericity was applied when appropriate. The *F* value, the uncorrected degrees of freedom, the probability level following correction, the *ε* value (when appropriate), and the *η^2^* effect size index are given. Statistically significant interactions were identified by tests of simple effects, with the Bonferroni correction being applied in order to control for the increase in type I errors.

*P1 component.* A repeated measures ANOVA was performed for the incongruent-congruent difference of mean amplitudes in the 100–150 ms window at occipital sites (O1, O2 and O3) taking *Laterality* (three levels from left to right) as the within-subject factor and *Group* (LMA and HMA) as the between-subjects factor.

The ANOVA showed no significant main effect or interaction (all *p* values above.27).

*N450.* A repeated measures ANOVA was performed for the incongruent-congruent difference of mean amplitudes in the 350–500 ms window at fronto-central (Fc1, Fcz, and Fc2) and central (C1, Cz and C2) sites taking *Frontality* (fronto-central and central) and *Laterality* (three levels from left to right) as the within-subject factor and *Group* (LMA and HMA) as the between-subjects factor. This time window was chosen based on previous literature and on the visual inspection of ERP waves.

**Figure 2:**
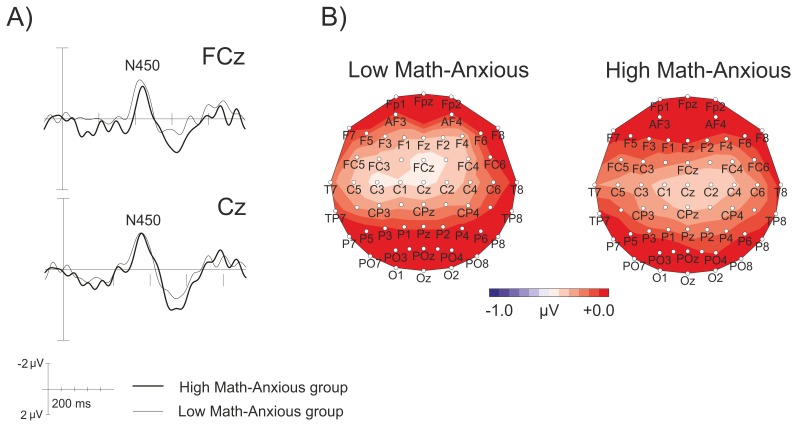
Grand average waveforms at FCz and Cz for the N450 component, showing the interference effect (incongruent-congruent) in the LMA and HMA groups (A); and the scalp topography of the N450 component, showing the interference effect in the 350–500 ms window for the LMA and HMA groups (B).

The ANOVA showed no *Group* significant main effect or interaction (all *p* values above.17). Figure 2 shows raw waves (A) and topographic maps (B) for the N450 component for the LMA and HMA groups, where the lack of differences between groups is shown. The mean amplitudes for N450 in the 350–500 ms window are shown in Table 3.

**Table 3:** Means and standard errors (in brackets) for N450 and Conflict-SP for conflict monitoring and conflict adaptation effects in the LMA and the HMA groups.

		Conflict monitoring	Conflict adaptation	
		Interference	Interference preceded by congruence	Interference preceded by incongruity
N450	LMA	−.67 (.16)	−.83 (.15)	−.13 (.19)
	HMA	−.53 (.14)	−.66 (.22)	−.64 (.24)
Conflict-SP	LMA	.50 (.10)	.38 (.18)	.41 (.20)
	HMA	.80 (.16)	1.16 (.28)	.25 (.19)

Note. Interference: incongruent – congruent; Interference preceded by congruence: (*cI-cC*); Interference preceded by incongruity: (*iI-iC*); N450: mean amplitude at Cz for the 350–500 ms window; CSP: mean amplitude at Cz for the 550–750 ms window.

*Conflict-SP.* A repeated measures ANOVA was performed for the incongruent – congruent differences of mean amplitudes in the 550–750 ms window at central sites (C1, Cz and C2) taking *Laterality* (three levels from left to right) as the within-subject factor and *Group* (LMA and HMA) as the between-subjects factor. This time window was chosen based on previous literature and on the visual inspection of ERP waves.

**Figure 3:**
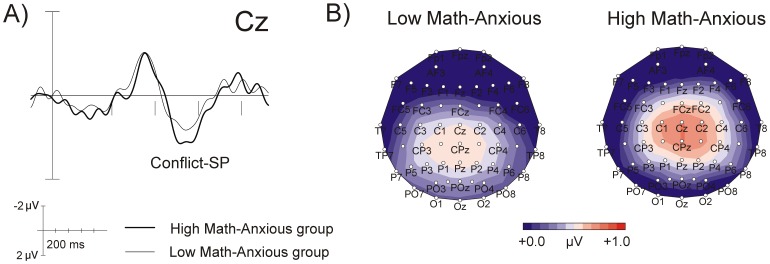
Grand average waveforms at Cz for Conflict-SP, showing the interference effect (incongruent-congruent) in the LMA and HMA groups (A); and the scalp topography of Conflict-SP, showing the interference effect in the 550–750 ms window for the LMA and HMA groups (B).

The overall ANOVA revealed a marginally significant main effect of *Group* (*F*(1,32) = 2.79, *p = *.09, *η^2^* = .08), with the HMA group showing a greater positivity (e.g., at Cz mean = .80 µV, SEM = .16) than the LMA one (mean = .50 µV, SEM = .10). Figure 3 shows raw waves (A) and topographic maps (B) for Conflict-SP for the HMA and LMA groups, showing greater amplitude for the HMA group as compared to the LMA one. The mean amplitudes for Conflict-SP in the 550–750 ms window are shown in Table 3.

**Conflict adaptation analysis.** *P1 component.* A repeated measures ANOVA was performed for the mean amplitude of the interference effect preceded by congruence (*cI-cC*) and preceded by incongruity (*iI-iC*) in the 100–150 ms window at occipital sites (O1, Oz and O2), taking *Previous congruence* (congruent and incongruent) and *Laterality* (three levels from left to right) as within-subject factors and *Group* (LMA and HMA) as the between-subjects factor.

The ANOVA showed no significant main effect or interaction (all *p* values above.24).

*N450.* A repeated measures ANOVA was performed for the mean amplitude of the interference effect preceded by congruence (*cI-cC*) and preceded by incongruity (*iI-iC*) in the 350–500 ms window at fronto-central (Fc1, Fcz, and Fc2) and central (C1, Cz and C2) sites, taking *Previous congruence* (congruent and incongruent), frontality (fronto-central and central) and *Laterality* (three levels from left to right) as within-subject factors and *Group* (LMA and HMA) as the between-subjects factor.

**Figure 4:**
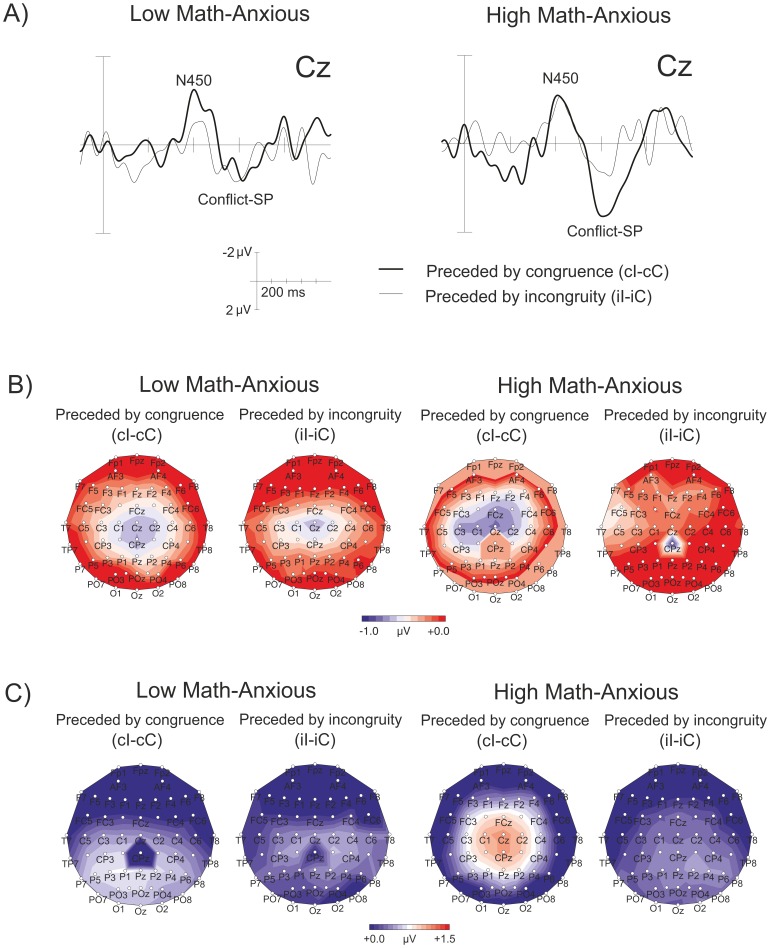
Grand average waveforms at Cz for the N450 component and for the Conflict-SP, showing the interference effect preceded by congruence (*cI-cC*) and by incongruity (*iI-iC*) for the LMA and HMA groups (A) the scalp topography of the N450 component, showing the interference effect preceded by congruence (*cI-cC*) and by incongruity (*iI-iC*) in the 350–500 ms window for the LMA and HMA groups (B) and the scalp topography of Conflict-SP, showing the interference effect preceded by congruence (*cI-cC*) and by incongruity (*iI-iC*) in the 550–750 ms window for the LMA and HMA groups (C).

The ANOVA showed a significant main effect of *Previous congruence* (*F*(1,32) = 5.61, *p = *.02, *η^2^* = .14), with the amplitude of N450 being more negative when preceded by congruence (mean = −.69, SEM = .11) than when preceded by incongruity (mean = −.33, SEM = .13). The *Group x Frontality* interaction was also significant (*F*(1,32) = 3.89, *p = *.05, *η^2^* = .10). In order to analyze this interaction, separate ANOVAS were performed at fronto-central and central sites. While no *Group* main effect or interactions emerged at fronto-central sites (all *p* values above.31), a significant *Group x Previous congruence* interaction (*F*(1,32) = 3.95, *p* = .05, *η^2^* = .11) was found at central sites. This interaction showed that for the LMA group, the N450 was more negative when preceded by congruence (mean = −.83 µV, SEM = .15) than when preceded by incongruity (mean = −.13 µV, SEM = .19) (*p* = .004), while no differences were found for the HMA group (*p* = .80). Figure 4 shows raw waves (A) and topographic maps (B) for N450 elicited for the interference effect preceded by congruence (*cI-cC*) and by incongruity (*iI-iC*) for the LMA and the HMA groups. This figure clearly shows a greater N450 component for the interference effect preceded by congruence than when preceded by incongruity only for the LMA group. The mean amplitudes for N450 in the 350–500 ms window are shown in Table 3.

*Conflict-SP.* A repeated measures ANOVA was performed for the mean amplitude of the interference effect preceded by congruence (*cI-cC*) and preceded by incongruity (*iI-iC*) in the 550–750 ms window at central sites (C1, Cz, and C2), taking *Previous congruence* (congruent and incongruent) and *Laterality* (three levels from left to right) as within-subject factors and *Group* (LMA and HMA) as the between-subjects factor.

The ANOVA showed a significant *Previous congruence* × *Group* interaction (*F*(1,32) = 4.20, *p = *.04, *η^2^* = .11), with a greater amplitude for the interference effect preceded by congruence (mean = 1.16, SEM = .28) than when preceded by incongruity (mean = .25, SEM = .19) for the HMA group (*p* = .01), but no differences for the LMA one (*p* = .84). Apart from the marginally significant main effects of *Previous congruence* (*F*(1,32) = 3.15, *p = *.08, *η^2^* = .09) and *Group* (*F*(1,32) = 3.22, *p = *.08, *η^2^* = .09), all the other effects and interactions were not significant (all *p* values above.15). Figure 4 shows raw waves (A) and topographic maps (B) for Conflict-SP for the interference effect preceded by congruence (*cI-cC*) and by incongruity (*iI-iC*) for the LMA and the HMA groups. The figure clearly shows that the HMA group showed a more positive amplitude for the interference effect preceded by congruence than when preceded by incongruity, while no differences emerged for the LMA group. The mean amplitudes for Conflict-SP in the 550–750 ms window are shown in Table 3.

### Correlational Analyses

#### Relation between math anxiety and behavioral measures

Participants’ scores on the sMARS test were correlated with the interference effect shown in behavioral measures for the conflict monitoring and conflict adaptation effects. Results are shown in Table 4. This table shows that the higher the level of math anxiety, the greater the interference in response times for the current trial and the greater the interference in response times when preceded by incongruity.

**Table 4:** Pearson correlation coefficients between the sMARS scores and behavioral measures for conflict monitoring and conflict adaptation for the whole sample (n = 34).

	Conflict monitoring		Conflict adaptation			
	Reaction time	Accuracy	Reaction time		Accuracy	
	Interference	Interference	Interference preceded by congruence	Interference preceded by incongruity	Interference preceded by congruence	Interference preceded by incongruity
sMARS	.34 *	−.05	.16	.37 *	−.09	−.01

Note. * *p*<.05.

#### Relation between math anxiety and ERP measures

The sMARS scores were also correlated with the mean amplitude of N450 and Conflict-SP for the conflict monitoring and conflict adaptation effects. Results are shown in Table 5. This table shows that the higher the level of math anxiety, the greater the amplitude of the Conflict-SP when preceded by congruence.

**Table 5:** Pearson correlation coefficients between the sMARS scores and ERP measures for conflict monitoring and conflict adaptation for the whole sample (n = 34).

	Conflict monitoring		Conflict adaptation			
	N450	Conflict-SP	N450		Conflict-SP	
	Interference	Interference	Interference preceded by congruence	Interference preceded by incongruity	Interference preceded by congruence	Interference preceded by incongruity
sMARS	.10	.30	.10	−.29	.42*	−.11

Note. * *p*<.05; N450: mean amplitude at Cz for the 350–500 ms; Conflict-SP: mean amplitude at Cz for the 550–750 ms window.

#### Relation between behavioral and ERP measures

Finally, ERP measures of conflict monitoring and conflict adaptation were correlated with the interference effect shown in behavioral measures for these effects. Results are shown in Table 6. This table shows that the greater the interference in hit rates (more errors committed in the incongruent condition than in the congruent one), the more negative the amplitude of the N450 and the more positive the amplitude of the Conflict-SP for the interference effect preceded by congruence.

**Table 6:** Pearson correlation coefficients between behavioral and ERP measures for conflict monitoring and conflict adaptation for the whole sample (n = 34).

Behavioral measures			ERP measures						
			Conflict Monitoring			Conflict Adaptation			
			N450	Conflict-SP		N450		Conflict-SP	
			Interference			Interference preceded by congruence	Interference preceded by incongruity	Interference preceded by congruence	Interference preceded by incongruity
Conflict Monitoring	Reaction time	Interference	.02		.13	.06	−.06	.22	.24
	Accuracy	Interference	−.30		.48*	−.36*	−.10	.43*	.03
Conflict Adaptation	Reaction time	Interference precededby congruence	.15		.002	.20	.03	.04	.24
		Interference precededby incongruity	−.11		.08	−.13	−.13	.23	.11
	Accuracy	Interference precededby congruence	−.34		.35	−.32	−.10	.43*	.003
		Interference precededby incongruity	.04		.50*	−.17	.009	.33	.05

Note. * *p*<.05; N450: mean amplitude at Cz for the 350–500 ms; Conflict-SP: mean amplitude at Cz for the 550–750 ms window.

## Discussion

This study aimed to investigate numeric conflict monitoring and conflict adaptation in high math-anxious individuals with the help of the ERP technique, in order to investigate further whether math anxiety is related to difficulties in early and/or later stages of conflict processing, and to better understand math anxiety-related differences in the execution of attentional control when conflict is encountered in processing. As far as we know, this is the first time that numeric conflict monitoring and adaptation are studied with ERPs in math anxious individuals. To this end, we formed two groups that were extreme in math anxiety, but that did not differ in trait anxiety, state anxiety or math ability, enabling us to rule out the possibility that the expected differences between groups could be attributed to these variables. Both groups had to solve a numerical Stroop task involving congruent and incongruent trials in equal proportion. We expected to reproduce previous research by finding a greater interference in response times for the HMA group. The ERP technique helped to identify two conflict-related ERP components enabling us to determine whether math anxiety is related to a first stage of conflict detection (i.e., N450) and/or to a later response-related (i.e., Conflict-SP) stage of conflict processing. Moreover, conflict adaptation analysis provides useful information regarding possible variations in attentional control in math anxious individuals depending on the congruence of the previous trial, as previously suggested for trait anxiety [45], [48].

Regarding behavioral measures, and consistent with previous studies in math anxiety, a greater interference effect was found in response times for the HMA group as compared to the LMA one [17], [23]. This corroborates the main claims of the ACT [9] arguing that high anxious individuals are characterized by a greater influence of the stimulus-driven attentional system relative to the goal-directed attentional system. In this way, according to this theory, HMA individuals would be more influenced by the distractor dimension of the stimuli (i.e., number size) interfering with the task-relevant dimension of the task (i.e., numerical magnitude), which would explain why they needed more time to solve trials presenting a stimuli-response conflict than their LMA counterparts. Also in this respect, we found a significant positive correlation between interference in response times and math anxiety; the greater the level of math anxiety, the more time needed to respond to incongruent trials as compared to congruent ones. Moreover, in accordance with the ACT [9] and the original PET [10], the effects of math anxiety were shown on response times (i.e., processing efficiency) but not on hit rates (i.e., performance effectiveness), given that anxiety is considered not to directly affect the level of performance on a task, but to reduce the efficiency with which the task is solved.

Regarding electrophysiological data, we were able to replicate the results of previous studies by identifying two ERP components crucially linked to stimulus-response conflicts in the Stroop task, namely, N450 and Conflict-SP. Our conflict monitoring analysis showed that math-anxious individuals did not differ in a first conflict detection stage of processing, given that there were no differences between groups for the N450 component (neither for an even earlier P1 component). However, the HMA group did show a tendency for greater Conflict-SP amplitude than the LMA group. It is not easy to say what this difference is telling us, given that this component has been related with a very wide range of cognitive processes such as general preparation [33], response selection [18], conflict processing [29], [60], and execution of top-down control [38]. Nevertheless, conflict adaptation analysis can help us to clarify the evidence on cognitive function signaled by this Conflict-SP, and thus to give support to one of these possible interpretations. Conflict adaptation effects were first reported by Gratton et al. (1992), who found that the interference effect was enhanced when preceded by congruent trials [40]. The conflict monitoring model explains this finding as an enhancement in attentional control when incongruity is found. If attentional control is enhanced in the previous trial, the task-irrelevant dimension of the stimulus has less influence, and thus the interference effect is reduced. We were able to replicate this effect in our data by finding larger response times and reduced hit rates for the interference effect preceded by congruence (which does not enhance attentional control) as compared to the interference effect preceded by incongruity (considered to enhance attentional control). Nevertheless, in line with previous evidence on trait anxiety, no significant group differences were obtained for these behavioral measures of conflict adaptation [48]. The reason may be that behavioral measures often provide very indirect evidence of internal processes such as cognitive control, which can sometimes only be detected using more sensitive techniques, such as ERPs.

In fact, ERPs showed differences in conflict adaptation between math anxious groups for the N450. More specifically, we found that while the LMA group showed a more negative N450 for the interference effect preceded by congruence than when preceded by incongruity, the HMA group showed no difference in this component in relation to the congruence of the previous trial. Previous evidence has shown that the N450 shows greater amplitudes when the level of conflict is higher [31]. Similarly, we found a negative correlation between the interference in hit rates and the amplitude of the N450 when preceded by congruence, showing that as the level of interference increased, the N450 became more negative. These results suggest that the LMA group experienced a higher level of conflict due to the interference effect preceded by congruence than when preceded by incongruity. In other words, while the LMA group showed the expected conflict adaptation effect pattern (i.e. greater interference when preceded by congruence), the HMA group did not show this effect at this first stage of conflict processing.

Previous evidence has suggested that the N450 component showed no variation with previous trial congruence [38]. Using a color-naming Stroop task in normal participants, Larson et al. (2009) found that the N450 component did not vary according to the congruence of the previous-trial, and they proposed that this component reflected neural processes that were more automatic, regardless of the amount of top-down control needed during a particular trial. In contrast, we found that the congruence of the previous trial did modulate the amplitude of this component in LMA individuals, suggesting that it is modulated by variations in attentional control, and therefore, that it reflects more than a simple automatic process [48]. Similarly, using a gender discrimination Stroop task with the help of the ERP technique, Osinsky et al. (2010) also found a modulation of the N450 amplitude with variations of the congruence of the previous trial for trait anxiety; more specifically, they obtained a greater N450 component for the interference effect preceded by incongruity than when preceded by congruence for the high trait anxious group [48]. They tentatively interpreted this finding as indicating a reactive engagement of the conflict monitor as a direct response to an acute need for top-down guidance. In contrast, we obtained a normal and expected conflict adaptation effect (greater N450 for the interference effect preceded by congruence) for the LMA group but no conflict adaptation at all for the HMA group.

Conflict adaptation analysis also showed very interesting effects for the Conflict-SP. More specifically, we found that, while no differences were obtained for the LMA group depending on the congruence of the previous trial, the HMA group showed greater Conflict-SP amplitude for the interference effect preceded by congruence than when preceded by incongruity. This result suggests that the tendency for greater Conflict-SP amplitude for the HMA group in the conflict monitoring analysis (current trial congruence effects) might be due to the greater amplitude for this component when it is preceded by congruence, while the interference effect preceded by incongruity shows a similar pattern for the LMA group. This result gives support to previous evidence suggesting that Conflict-SP reflects controlled processes that adapt to the level of control necessary to accurately complete the trial [38]. Moreover, a significant positive correlation emerged between math anxiety scores and Conflict-SP for the interference effect preceded by congruence, showing that the higher the level of math anxiety, the greater the amplitude at this later stage of conflict processing.

These results give support to the DMC account, suggesting that high anxious individuals are characterized by a tendency to exert attentional control in a reactive way, that is, only when conflict is encountered in processing. On the other hand, low anxious individuals are considered to exert attentional control in a proactive way, by maintaining task goals over time. Previous investigations have given support to this account. For example, Fales et al. (2008) carried out a mixed blocked/event-related fMRI design to track transient (i.e. reactive) and sustained (i.e. proactive) activity in the dorsolateral prefrontal cortex (DLPFC) (an area considered to support cognitive control) while high and low anxious participants performed a working memory task. Results showed that high and low anxious individuals made strikingly different use of cognitive and default-network circuitry during the performance of a cognitive task. More concretely, they reported a positive correlation between trait anxiety and transient (i.e. reactive) activation of the DLPFC during working memory performance [47]. Similarly, using a gender discrimination task including congruent and incongruent face-word pairings and incorporating stimuli presenting only the task-relevant (face) and the task-irrelevant (word) dimensions of the stimuli, Osinsky et al. (2012) found that after incongruent trials, high trait-anxious individuals showed higher processing of the task-relevant dimension of the stimulus and suppressed processing of the task-irrelevant dimension of the stimulus, which also suggested a conflict-driven reactive recruitment of cognitive control in high trait-anxious individuals [45]. Our study, by finding that HMA individuals only exert attentional control after incongruent trials (Conflict-SP showed enhanced amplitude for the interference effect preceded by congruence), extends these findings to the field of math anxiety.

Moreover, according to the DMC model, this difference in the way attentional control is exerted depending on the level of anxiety has consequences on the susceptibility to distraction. In this way, HMA individuals, by exerting attentional control only when conflict is encountered in processing, would be more easily influenced by bottom-up input (i.e., the ACT’s stimulus-driven attentional system) [13], and thus would be more easily distracted. On the other hand, LMA individuals, by sustaining task requirements or goals over time, would show more effective top-down control of processing (i.e., the ACT’s goal-directed attentional system) [13] and thus would be less influenced by distraction. Consequently, the greater interference effect found for response times in the HMA group might be explained by differences in the way attentional control is exerted, by making HMA individuals more vulnerable to task-irrelevant information.

Two important aspects of this study deserve mention. The state anxiety measure we reported in the Participants section was obtained during the screening phase of this study (and not after the experimental task performed in the lab). The STAI was always administered after the math ability and the sMARS tests. Despite going through these math-related situations, the LMA and HMA groups did not differ in terms of their state-anxiety scores. However it might still be the case that they differed during the experimental task, and so we cannot rule out the possibility that our results show some effect of state anxiety apart from the effect of (trait) math anxiety. Second, beyond the congruence effect generated by presenting pairs of numbers showing a conflict between numerical magnitude and physical size, number pairs also differed in their distance from each other, i.e. being close (distance 1; e.g. 1–2 and 8–9) or further away (distance 7; e.g. 1–8 and 2–9). Conceivably, it could be that the distance effect introduced some undesired variability in our data. However, an additional analysis was performed for response times to test this possibility, and the results showed the expected *distance effect* in our data, distance 1 requiring more time than distance 7, but this effect did not affect the two groups in different ways (no significant group main effect or interaction emerged), suggesting that this effect cannot explain our findings.

Although our math anxious individuals did not differ in their conflict monitoring (only considering the effect of the current trial), they showed very interesting differences in their responses and adaptation to the congruence of the previous trial. LMA individuals showed a conflict adaptation effect in the first stage of conflict processing (N450) followed by a proactive execution of attentional control, which was exerted for the interference effect preceded both by congruence and by incongruity. In contrast, high math-anxious individuals were characterized by an absence of a conflict adaptation effect in the first stage of conflict processing followed by a reactive and compensatory recruitment of control resources and goal-directed attention, which was exerted only when they had previously been exposed to stimuli presenting conflicting information. In view of previous evidence claiming that a reactive execution of attentional control contributes to a greater susceptibility to distraction, and given that, in our study, this lack of enhancement in attentional control after congruent trials was related to a failure to overcome conflict (i.e. after congruence, the greater the Conflict-SP amplitude, the greater the interference in accuracy), this difference in the execution of attentional control after conflict detection may very well explain the differences between low and high math-anxious individuals when processing numerical conflict.

As far as we know, this study is the first attempt to identify the electrophysiological correlates of conflict monitoring and conflict adaptation in math anxious individuals, while controlling for general anxiety and math ability. We have replicated previous studies showing greater numeric interference in response times for the HMA group, suggesting that math anxiety affects higher-order functions of cognitive control, making task-irrelevant information more intrusive for this group as compared to the LMA one [17], [23]. It is worth mentioning that, in our study, HMA individuals showed greater susceptibility to distraction in a task involving conflict between numerical magnitude and physical size. Nevertheless, this susceptibility to distraction is not limited to this kind of information, but also extends to the distractor effect that internal stimuli, such as worrying thoughts and ruminations, have on working memory [9]. As a consequence, HMA individuals may also be more vulnerable to these kinds of thoughts that attract attention away from the task and impair performance. The effects of distraction could be especially detrimental in the learning of mathematics, given its cumulative nature, one concept building on the next. For this reason, attentional control deficit and distractibility in high math anxious individuals constitutes a key aspect deserving further research.
